# Beneficial Services of Arbuscular Mycorrhizal Fungi – From Ecology to Application

**DOI:** 10.3389/fpls.2018.01270

**Published:** 2018-09-04

**Authors:** Min Chen, Miguel Arato, Lorenzo Borghi, Eva Nouri, Didier Reinhardt

**Affiliations:** ^1^Department of Biology, Rte Albert Gockel, University of Fribourg, Fribourg, Switzerland; ^2^Inoq GmbH, Schnega, Germany; ^3^Institute of Plant and Molecular Biology, University of Zurich, Zurich, Switzerland

**Keywords:** arbuscular mycorrhiza, symbiosis, abiotic stress, plant growth, plant protection, plant nutrition, soil structure, Glomeromycota

## Abstract

Arbuscular mycorrhiza (AM) is the most common symbiotic association of plants with microbes. AM fungi occur in the majority of natural habitats and they provide a range of important ecological services, in particular by improving plant nutrition, stress resistance and tolerance, soil structure and fertility. AM fungi also interact with most crop plants including cereals, vegetables, and fruit trees, therefore, they receive increasing attention for their potential use in sustainable agriculture. Basic research of the past decade has revealed the existence of a dedicated recognition and signaling pathway that is required for AM. Furthermore, recent evidence provided new insight into the exchange of nutritional benefits between the symbiotic partners. The great potential for application of AM has given rise to a thriving industry for AM-related products for agriculture, horticulture, and landscaping. Here, we discuss new developments in these fields, and we highlight future potential and limits toward the use of AM fungi for plant production.

## Introduction

If an innovation spreads globally, becomes adapted to a multitude of diverse applications and persists over eons of time, it can be considered a great success. This is certainly the case for arbuscular mycorrhiza (AM). AM is thought to have a monophyletic origin in the Ordovician, approximately 480 Mio years ago (Redecker et al., 2000; Delaux, 2017), and it is found in the majority of land plants in most taxa and virtually all ecological niches (Read, 2002; Wang and Qiu, 2006). Most land plants are facultative symbionts, i.e., they profit from AM fungi, but can also live without them, although at considerable fitness costs (see below). However, some plant species have turned to obligate parasites on the AM fungus, i.e., they became fully dependent on fungal nutrition and lost photosynthetic capacity (mycoheterotrophs) (Graham et al., 2017). On the other end of the scale, some plant taxa, e.g., the *Brassicacea* and *Chenopodiaceae*, became asymbiotic, i.e., they lost the capacity to interact with AM fungi and evolved alternative strategies to meet their nutritional needs (Brundrett, 2004).

Arbuscular mycorrhiza symbiosis is thought to be a largely promiscuous association between >100,000 plant species and a few 100 AM fungal morphotypes, which have long been regarded as the equivalent of species. However, due to the relatively few distinctive morphological features of AM fungi (primarily associated with spores), and due to their essentially asexual mode of propagation, the traditional species concept is problematic in the context of AM fungi. AM fungi have never been shown to form sexual stages or to mate, however, they can undergo hyphal fusion (anastomoses) and exchange genetic material, thereby reshuffling their genomes and generating new genetic diversity in the absence of classical meiosis and recombination (Chagnon, 2014). Anastomosis depends on genetic relatedness, hence this feature could potentially be used as an additional criterion to define taxonomic units besides spore morphotypes.

With the advent of large scale sequencing approaches, AM fungal taxonomy and systematics rose to a new level (Spatafora et al., 2016). Results obtained with these modern tools indicate that the diversity of AM fungi has been underestimated (Husband et al., 2002; Öpik et al., 2006, 2013; Lee et al., 2013). Hence, the true number of AM fungal species, including genetically and functionally distinct “cryptic species” that cannot be distinguished by morphometric parameters (Munkvold et al., 2004; Rosendahl, 2008; Chen et al., 2018; Savary et al., 2018), may exceed current estimates by orders of magnitudes. The fact that recent results have documented unprecedented genetic variability even within one AM fungal species at a given site (Chen et al., 2018) points to the fact that the peculiar genetics and mode of reproduction of AM fungi impede with systematics and nomenclature in AM fungi.

## Origin and Evolution of AM

Recent evidence indicates that the evolution of early plants from non-photosynthetic eukaryotes occurred in a freshwater environment by engulfment and domestication of a photosynthetic cyanobacterium (which subsequently evolved to the chloroplasts) (Ponce-Toledo et al., 2017). Hence, plants are the result of an endosymbiosis that was successful enough to allow them to radiate through most aquatic environments. Which innovations allowed plants to subsequently conquer the dry land masses of the continents? Some of the obvious adaptations required for the colonization of this new environment include protection against high radiation, a water-impermeant cuticle, and water-conductive vascular systems. However, an equally important innovation was required to allow plants to acquire water and nutrients from the substrate in the absence of specialized absorptive organs such as roots, which only evolved later (Brundrett, 2002). Conceivably, fungal symbioses were instrumental to allow the colonization of land by descendants of freshwater algae (Bidartondo et al., 2011; Delwiche and Cooper, 2015; de Vries and Archibald, 2018).

Although associations with AM fungi may not have been the first fungal symbiosis of early land plants (Field et al., 2015), recent evidence suggests that the advent of AM in the early land plants was a unique event, hence, AM appear to be a monophyletic innovation that may have enabled the rapid colonization of the continents by vascular plants (Delaux, 2017). Thus, it is conceivable that early rootless plants engaged in various kinds of fungal associations, as they are still observed today in early-diverging plant lineages (Read et al., 2000), and that roots coevolved with AM in the vascular plants (Brundrett, 2002). AM fungal associations were so successful that still the majority of land plants in most ecological niches (except for aquatic environments) engage in this symbiotic association.

## Mechanisms Involved in Intracellular Accommodation of AM Fungi

The very long evolutionary history of AM symbiosis of more than 400 Mio years (Redecker et al., 2000; Heckman et al., 2001; Schüssler et al., 2001), and the involvement of plant-derived and fungal signaling molecules that promote AM (Gutjahr and Parniske, 2013), suggests a high degree of adaptation and genetic/metabolic coordination between mycorrhizal partners. Indeed, formation of AM requires a dedicated signaling pathway starting with the root-borne signal strigolactone, which is exuded to stimulate AM fungal activity (Akiyama et al., 2005; Besserer et al., 2006; Kretzschmar et al., 2012). AM fungi subsequently secrete lipochito-oligosaccharides, which are perceived by the plant and activate a signal transduction pathway that is shared with root nodule symbiosis and therefore is known as the common symbiosis signaling pathway (CSSP), which has been elucidated in great detail in recent years (Harrison, 2012; Gutjahr and Parniske, 2013). In the light of the very low host specificity in AM, the involvement of a bidirectional exchange of symbiosis signals challenges our current understanding of communication between the partners, since it would require either many alternative signals for each potential partner, or few signals that can be recognized by a wide range of potential partners.

While central questions related to recognition and infection remain open, a rich body of microscopic evidence shows that at later stages the interaction has a very high degree of coordination at the cellular level. The most impressive examples are the formation of an infection structure (prepenetration apparatus; PPA) that allows cellular invasion (Genre et al., 2005, 2008), and the formation of the intracellular arbuscules that serve as nutritional interface between the partners (Harrison, 2012; Gutjahr and Parniske, 2013). Although the molecular-genetic basis of PPA formation is elusive, PPAs are thought to be a prerequisite for AM fungal infection of host roots, and to require signaling through the CSSP (Genre et al., 2005). Establishment of AM is associated with a fundamental reprogramming of the host cells including the activation of hundreds of genes (Liu et al., 2003; Güimil et al., 2005; Hohnjec et al., 2005; Fiorilli et al., 2009; Gomez et al., 2009; Guether et al., 2009; Breuillin et al., 2010; Gaude et al., 2012; Tromas et al., 2012; Hogekamp and Küster, 2013; Calabrese et al., 2017), of which some are expressed primarily or exclusively in cells with arbuscules. Although these genes are thought to be required for intracellular accommodation of the fungus, and for coordination of symbiotic functions, their molecular and cellular function has been elucidated only in few cases (see below).

## New Paradigms in the Exchange of Benefits in AM Symbiosis

**FIGURE 1:**
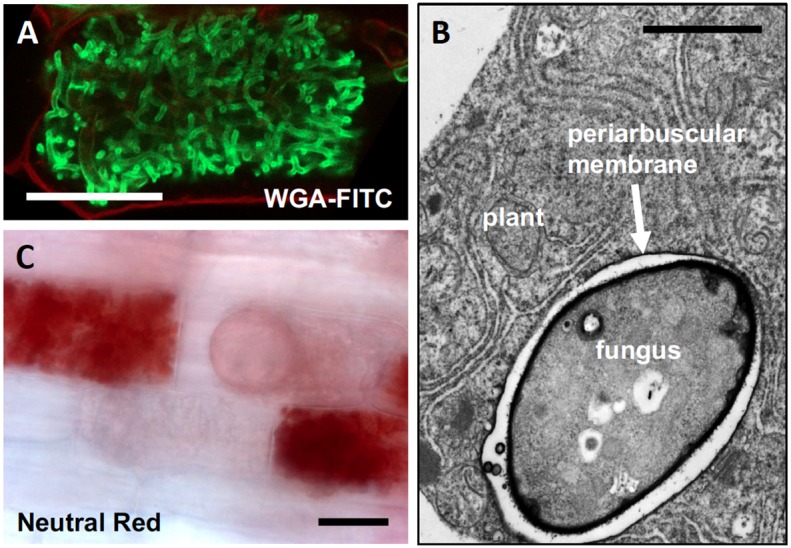
Characteristics of AM fungal arbuscules. **(A)** Arbuscules are highly branched hyphal structures that nearly fill the cortex cells of the host. Green staining of fungal structures with wheat germ agglutinin (WGA) coupled to fluorescein isothiocyanate (FITC), red staining of the cell wall with propidium iodide (from Kretzschmar et al., 2012). **(B)** Transmission electron micrograph of a colonized host cell with an arbuscular branch (fungus), surrounded by the periarbuscular membrane. **(C)** A colonized root stained with Neutral Red which accumulates in acidic compartments, in this case the space between the periarbuscular membrane and the fungal cell wall [compare with **(B)]**. Size bars, 20 μm in **(A,C)**; 1 μm in **(B)**.

The finely branched fungal arbuscules (**Figure 1A**), and the surrounding peri-arbuscular membrane of the host (**Figure 1B**), represent a considerably increased contact surface (also known as symbiotic interface) between the two partners, which has been estimated to correspond to a multiple of the entire cell surface (Alexander et al., 1989). In addition, the symbiotic interface is acidified (**Figure 1C**) to energize nutrient transport across the fungal plasma membrane and the periarbuscular membrane (Guttenberger, 2000; Krajinski et al., 2014; Wang et al., 2014). Therefore, cells with arbuscules are ideally suited for nutrient exchange. Indeed, the plant host expresses many symbiosis-specific nutrient transporters that are thought to mediate mineral nutrient uptake from the AM fungus (Rausch et al., 2001). The best-characterized example is a symbiotic phosphate transporter (PT) that is expressed exclusively in cells with arbuscules (MtPT4 in *Medicago truncatula*; OsPT11 in rice) (Harrison et al., 2002; Yang et al., 2012). Phylogenomic analysis of MtPT4 and its orthologs in other land plants suggests that the AM-related phosphate uptake pathway represents an early evolutionary innovation that became conserved after the advent of the angiosperms (Vigneron et al., 2018). Phosphate delivery is among the most important benefits for the host in AM symbiosis (Karandashov and Bucher, 2005), and the collective information suggests that the arbuscules are the site of transfer of phosphate from the fungus to the plant (MacLean et al., 2017).

The induction of many other mineral nutrient transporters in mycorrhizal roots (Wang et al., 2017), and the fact that mycorrhizal plants contain increased amounts of various mineral nutrient elements (Clark and Zeto, 2000; George, 2000) suggest that nutrient elements such as nitrogen, sulfur, and microminerals such as copper and zinc may also be transferred via the arbuscules. However, for most AM-induced predicted nutrient transporters, the expression pattern, protein localization, and function remain to be established.

Interestingly, AM-related pathways can also stimulate plant growth and physiology in nutrient-independent ways. For example, mycorrhizal plants show enhanced photosynthetic capacity (Boldt et al., 2011). More strikingly, the overexpression of a petunia strigolactone transporter (PDR1), which is involved in AM signaling (Kretzschmar et al., 2012), is sufficient to improve root and shoot growth in the absence of AM fungi (Liu et al., 2018). Thus, AM and its signaling can potentially increase plant growth in yet unexplored ways that are more related to plant developmental programs than to plant nutrition.

As a reward for its symbiotic services, the AM fungus receives fixed carbon from the plant. In analogy to plant pathogen interactions, carbon transfer has long been thought to proceed in the form of carbohydrates (in particular hexoses). Indeed, a large body of evidence has demonstrated that AM fungi can take up and utilize sugars, but only under symbiotic conditions in the roots (Roth and Paszkowski, 2017). Recently, the surprising discovery that two AM fungal genomes lack a fatty acid synthase complex (Wewer et al., 2014; Tang et al., 2016) has raised the question how AM fungi may generate their abundant lipid reserves in spores and vesicles (Rich et al., 2017b). Intriguingly, the plant host induces several components of fatty acid biosynthesis and processing in mycorrhizal roots indicating that AM fungi may also receive fatty acids besides sugars. Indeed, recent evidence has demonstrated that AM fungal lipids are, at least partially, derived from the plant host (Bravo et al., 2017; Jiang et al., 2017; Keymer et al., 2017; Luginbuehl et al., 2017; Brands et al., 2018).

The supply of lipids to AM fungi involves host genes encoding enzymes of fatty acid biosynthesis, a glycerol-3-phosphate acyl transferase (GPAT) that generates a monoacylglycerol (MAG) intermediate and a pair of ATP-binding cassette transporters of the G-type (ABCGs) that form a heterodimeric transporter in the peri-arbuscular membrane (Zhang et al., 2010). These elements resemble components required for the generation and secretion of the lipid precursor for the extracellular lipid polyester cutin, suggesting that the two pathways may share a common evolutionary origin in early land plants (Rich et al., 2017b). The AM-specific transcription factor REQUIRED FOR ARBUSCULAR MYCORRHIZA1 (RAM1) in the host is responsible for induction of many of the genes required for a functional AM, including the GPAT RAM2 and the ABCGs STUNTED ARBUSCULE (STR) and STR2 (Park et al., 2015; Rich et al., 2015; Pimprikar et al., 2016; Luginbuehl et al., 2017; Rich et al., 2017a). However, many aspects of lipid transfer to AM fungi remain to be elucidated.

## Significance of AM for Plants in Natural Habitats

How much a plant benefits from AM fungal colonization depends to a large degree on the environmental conditions. In most natural environments, which are characterized by mineral nutrient deficiency and various abiotic stress conditions, mycorrhizal plants are thought to have a selective advantage over non-mycorrhizal individuals of the same species. Thus, AM can potentially promote intraspecific competitiveness and selectively favor mycorrhizal plants. Conceivably, this is the reason why AM symbiosis has prevailed over very long periods of evolutionary time in most land plant taxa.

A complication arises due to the fact that plants can have several different AM fungal partners, and *vice versa*, each fungal mycelium can infect several host plants of the same or different species. The resulting common mycorrhizal networks (CMNs) add an additional level of complexity to the analysis of benefits in mycorrhizal interactions (Jakobsen and Hammer, 2015). A strongly interconnected plant community can potentially gain stability because weaker individuals could profit from mineral nutrient supply from the CMN at the expense of stronger plants that entertain the CMN. In this way, the stronger plants indirectly benefit less competitive plants, thereby attenuating competition among plant individuals. Such “underground socialism” has been envoked particularly in cases where seedlings grew better when they were connected to a CMN that had been established by older plants, a phenomenon known as facilitation (van der Heijden and Horton, 2009). However, the effects of CMN on seedlings are highly context-dependent and vary with the involved species. In some cases, AMF can even increase intra- or interspecific competition, hence, the effects of CMN cannot be generalized. In the most extreme version of the theme, achlorophyllous plants obtain all their resources, including carbon, from CMN, thereby parasitizing—indirectly—on other plants that supply the network with their carbon (Bidartondo et al., 2002). While this represents an extreme nutritional strategy that emerged only in a minority of land plants, there are many intermediate examples of plants that obtain part of their carbon from mycorrhizal fungi (mixotrophy), a condition that has likely been the transitional evolutionary phase from autotrophy to mycoheterotrophy (Bidartondo, 2005; Selosse et al., 2017).

## Functional Specificity in AM Interactions

**FIGURE 2:**
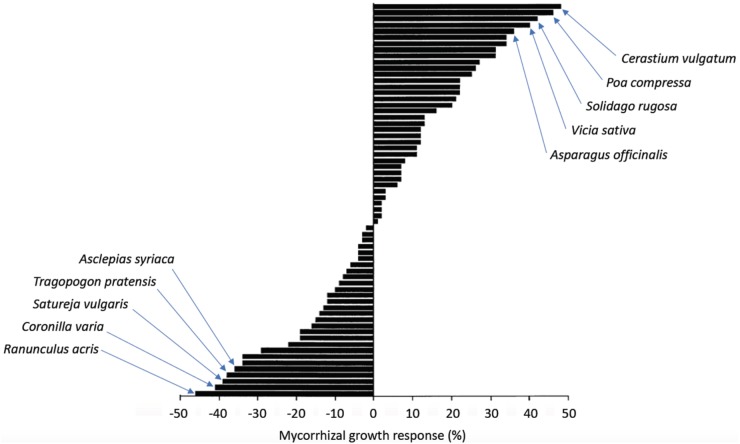
Mycorrhizal growth response (MGR) depends on the symbiotic partners. Various plants were inoculated with *G. etunicatum*. After 16 weeks of coculture, the dry weight of the host plants was determined and the percent change relative to the non-mycorrhizal controls (referred to as MGR) was calculated. MGR ranged between –50% and +50% change in dry weight. The 10 most responsive plants (five positive, five negative) are indicated (modified from Klironomos, 2003).

The variability of the effects of AM fungi on their hosts (see above) indicates that certain combinations are beneficial for the plant, whereas others are neutral or even negative. Conversely, AM fungal proliferation and sporulation are highly dependent on plant host identity (Bever, 2002). These findings suggest a certain degree of functional specialization in AM interactions. Indeed, a systematic combinatorial study on mycorrhizal benefits employing a large panel of plant and fungal species from different geographical locations showed that the mycorrhizal growth response (MGR; defined as the difference between the weights of mycorrhizal vs. non-mycorrhizal plants) ranged from -50% to +50% growth promotion, with almost half of the combinations resulting in growth depression (**Figure 2**) (Klironomos, 2003). The mutualistic potential did not correlate with phylogenetic patterns in either partner, indicative of adaptive mechanisms independent from lineage. Interestingly, combinations of partners isolated from the same location performed better, indicative of co-adaptation. Conceivably, combinations of good mutualists enjoy positive bidirectional feedback that results in progressive mutual adaptation of the most effective mutualistic combinations (Kiers and Denison, 2008), although the interaction shows very little host specificity at the level of infection (see above). In agreement with functional specialization, soils with a diverse AM fungal flora can support more diverse plant communities than if only one or few AM fungi are present (van der Heijden et al., 1998). This finding is compatible with a scenario in which each plant species requires a suitable AM fungal partner. Thus, despite the very low host specificity of AM under laboratory conditions, functional specialization within the AM fungal community shapes the level of the biodiversity and productivity of plant communities.

## Effects of AM Fungi on Plant Defense and Disease Resistance

Mycorrhizal roots often exhibit very intense fungal colonization, both intercellularly and intracellularly, that can reach more than 90% total root length. This observation has led Dangeard to coin the genus name *Rhizophagus* (greek for “root eater”), based on the initial assumption that mycorrhizal roots were colonized by an aggressive pathogen (Dangeard, 1900). We now know that most plants can potentially profit from AM fungal colonization (depending on the right fungal partner and the environmental conditions), but it is still a mystery how plants can tolerate such high degrees of colonization without mounting a defense response, given that fungi in general (including AM fungi) contain and release many molecular signals (e.g., chitin oligomers) that can be recognized by plants, and that have shown to trigger defense responses in various plant species (Wan et al., 2008; Boller and Felix, 2009). It has therefore been proposed that AM involves the suppression of defense. Indeed, plant mutants defective in genes required for symbiotic signaling and AM establishment (see above) often show characteristic defense responses upon infection by AM fungi, indicating that these fungi have potent signaling molecules that trigger defense, and that these mechanisms are suppressed during normal AM development. Pathogens usually produce inhibitors of defense (known as effectors), and recently, numerous effectors where also predicted to occur in the genomes of AM fungi (Sedzielewska Toro and Brachmann, 2016; Kamel et al., 2017). However, only very few of them have been functionally analyzed (Kloppholz et al., 2011).

Although defense mechanisms in the host have to be attenuated to allow AM fungal infection and colonization of the roots, general defense needs to remain active to cope with rhizospheric pathogens. Indeed, general disease resistance of mycorrhizal plants is not decreased. In contrast, mycorrhizal plants often exhibit increased disease resistance (Borowicz, 2001; Pozo and Azcon-Aguilar, 2007; Jung et al., 2012; Cameron D.D. et al., 2013). Experiments with split root systems revealed that this effect is often systemic, i.e., the entire plant is protected against pathogens. This can involve generally improved plant health due to better nutrition, or a systemic induction of the defense status, known as systemic acquired resistance (SAR). In addition, mycorrhizal plants may be prepared to react faster and stronger to pathogen attack, a phenomenon known as induced systemic resistance (ISR), or priming (Conrath et al., 2006). These protective effects of AM are of great interest for sustainable strategies of plant protection (Solaiman et al., 2014). Although priming is a systemic phenomenon, AM fungi are primarily employed to protect plants from soil-borne pathogens (Cameron D.D. et al., 2013; Jung et al., 2012). In addition, AM fungi, or other microbes associated with their mycelium, can directly interfere with rhizospheric pathogens either by the release of antimicrobial compounds, or by direct competition for space and resources. Although the potential of AM fungi for plant protection is widely acknowledged, it should be noted that in certain cases, mycorrhizal crops have no benefits from AM, or may even exhibit reduced growth and fitness (Jacott et al., 2017) (see also above). It is tempting to speculate that this phenomenon may be related to breeding programs that targeted traits related to shoot architecture and yield, while root-related traits were ignored. While this does not necessarily prevent plants from becoming infected, it may have interfered with the regulatory mechanisms that ensure optimal metabolic coordination of both partners.

## Significance of AM in the Major Climatic Zones and in Managed Ecosystems

Arbuscular mycorrhiza fungi have been observed in virtually all major ecosystems worldwide (Öpik et al., 2006), from arctic regions (Varga et al., 2015), to tropical forests (Lovelock et al., 2003), from the deserts in the arabic peninsula (Al-Yahya’ei et al., 2011) to the high himalayans (Liu et al., 2011). While some AM fungal isolates show only restricted distribution in natural communities, others appear to be true cosmopolitans (Rosendahl et al., 2009). Whether this reflects natural distribution, or transport by human activity is unclear. In addition, some cosmopolitan species may in fact represent genetically differentiated species complexes that cannot be distinguished by morphological criteria. The occurrence of truly cosmopolitan AM fungal species (Rosendahl et al., 2009) suggests that these fungi are extremely adaptable, both, in terms of environmental conditions, and in terms of a wide host range. Since AM fungi play an instrumental role in the protection against abiotic stresses such as nutrient starvation (see above), heat (Bunn et al., 2009), and drought (Augé, 2001; Ruiz-Sanchez et al., 2011; Rapparini and Penuelas, 2014; Chitarra et al., 2016), they can benefit their hosts in the wild and in agriculture (Wu, 2017). Consequently, AM fungi are thought to have a great impact in natural environments (Read, 2002; Smith and Read, 2008; van der Heijden et al., 2015), as in managed conditions in agriculture, horticulture, and forestry (see below).

## Reduction of Soil Erosion and Nutrient Leaching by AM

An important service of AM fungi in natural as well as in agricultural contexts is the beneficial alteration of soil structure (Leifheit et al., 2014). The dense hyphal network of the highly ramified AM fungal mycelium creates a three-dimensional matrix that enmeshes and crosslinks soil particles without compacting the soil. A soil glycoprotein was identified as an additional important agent in the stabilization of soil aggregates (Rillig, 2004; Singh et al., 2013). It is referred to as glomalin, because it is thought to be produced by AM fungi. Glomalin is not a defined gene product or chemically homogenous molecular species, rather, it is a soil fraction that is defined by its extractability and immuno-reactive properties (Rillig, 2004). Glomalin and glomalin-related soil proteins (GRSPs) have recently seen a renaissance in the literature, however, their origin and function are far from clear. Nevertheless, they represent an important determinant of soil quality and a very stable carbon sink with estimated half-life times in the range of several years up to decades (Rillig et al., 2001). GRSPs can account for a significant fraction of total organic soil carbon (2–5%), and since they protect other forms of organic carbon from degradation by increased soil particle aggregation, they may contribute significantly to sequestration of carbon in the soil (Rillig et al., 2001; Wilson et al., 2009). Taken together, the hyphal network of AM fungi, and their promoting effects on plant growth and root system development (Gutjahr and Paszkowski, 2013) protects the soil from erosion by wind and water.

The collective effects of AM fungi on soil qualities also results in higher water retention capacity, which benefits plant growth in addition to improved nutrient supply. The benefits of AM fungi are particularly critical for plants in dry sandy soils in arid regions. These soils often show low fertility and are highly vulnerable to erosion by wind and rain. In such cases, plantings with mycorrhizal plants can be a sustainable way to counteract erosion and improve soil fertility (see below).

Apart from the improved soil structure, AM fungi reduce nutrient leaching from the soil (Cavagnaro et al., 2015). Nutrient leaching is a serious problem since it results in loss of soil fertility and pollution of ground water and surface water (rivers, lakes). Intact ecosystems exhibit a good nutrient retention capacity due to efficient adsorption and retention of nutrients by roots and soil microorganisms (including AM fungi). However, agricultural soils are by definition disturbed by agricultural practice (in particular plowing), and they receive large amounts of fertilizer, mainly N, P, K. These, in particular the highly mobile nitrate, are prone to be washed out from the soil due to the lack of a good nutrient retention system (Cameron K.C. et al., 2013).

The beneficial effects of AM fungi against nutrient leaching operate at different levels. First, improved soil structure (see above) allows for increased nutrient sequestration to the micro- and macro-aggregates in mycorrhizal soil, second, AM fungi take up nutrients from the soil solution (Clark and Zeto, 2000; George, 2000), and final, mycorrhizal soils exhibit better retention capacity of the soil solution (see above) (Querejeta, 2017), thereby benefitting at the same time the availability of nutrients and water to the plant. A detailed documentation of the beneficial effect of AM fungi on plants under drought stress was reported for tomato (Bitterlich et al., 2018). Reduced leaching from mycorrhizal soils has been documented in particular for P and N, but it conceivably also involves other mineral nutrients. Taken together, AM fungi integrate the nutrient fluxes in the soil by generating closed nutrient cycles, thereby promoting long-term soil fertility (Cavagnaro et al., 2015).

## Commercial Use of AM Fungi

The multiple benefits of AM have raised opportunities for their commercial application. Consequently, the AM-related markets grew considerably during the past decades, with increasing numbers of actors, products and market volume (Vosatka et al., 2008). However, due to the fact that most of the AM-related industry consists of privately owned relatively small firms, public information about the dynamics of market shares are scarce. Hence, we carried out a systematic survey on the number of firms producing and selling AMF products in Europe and worldwide, and we assessed the number of their products as key figures in the market.

The results show that since the 1990s, the number of companies selling mycorrhizal products has increased considerably. On a global scale, the main players are located in North America, Europe, Asia, and Latin America. In the domain of the Americas, the main markets include United States, Canada, Mexico, Brazil, Argentina, Colombia, and Chile. The Asia region is mainly dominated by India, followed by China. The Indian market itself has seen an outstanding growth rate during the last decade. One of the reasons is the promotion of mycorrhiza-based bio-stimulants by the Indian government and the actions from organizations such as The Energy and Resources Institute (TERI^1^). In general, the AMF businesses are small- and medium-sized firms producing for the local and regional markets. However, there are some exceptions of larger companies from the United States, Canada, Germany, Italy, Czech Republic, United Kingdom, and Spain that export their products to various geographical regions.

**FIGURE 3:**
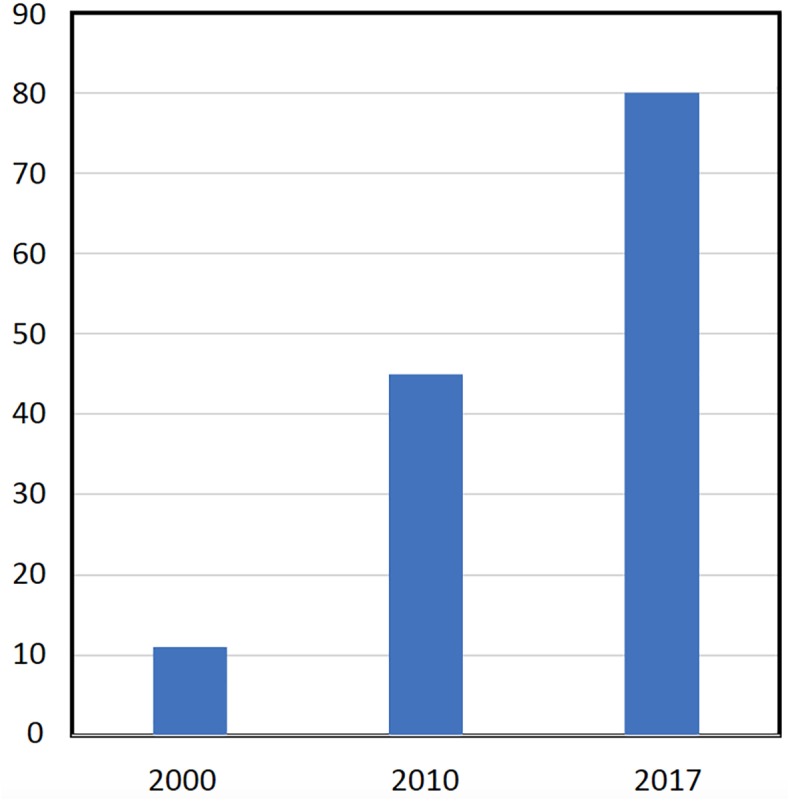
Increase in the number of companies in the European AM market. A survey on the number of firms selling AM inocula in Europe was determined by an internet surveyed. Based on the year of foundation, the number of firms was determined for three time points (year 2000, 2010, 2017).

**FIGURE 4:**
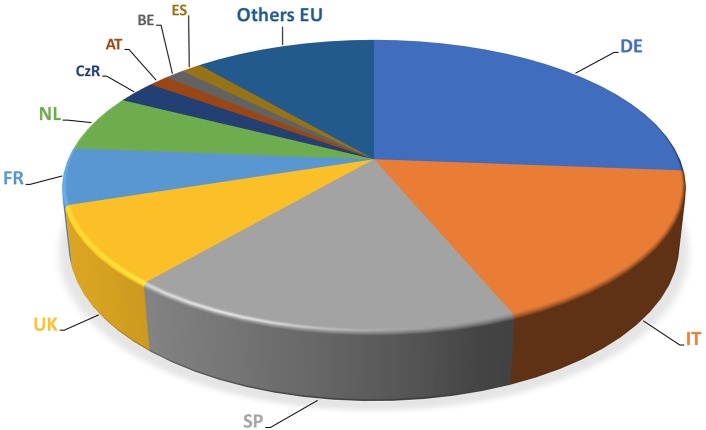
Main players in the AM market in the European Union. The number of companies selling AM inocula is expressed in relation of their location of the main house. Main producer countries are Germany (DE), Italy (IT), Spain (SP), the United Kingdom (UK), France (FR), and the Netherlands (NL).

**FIGURE 5:**
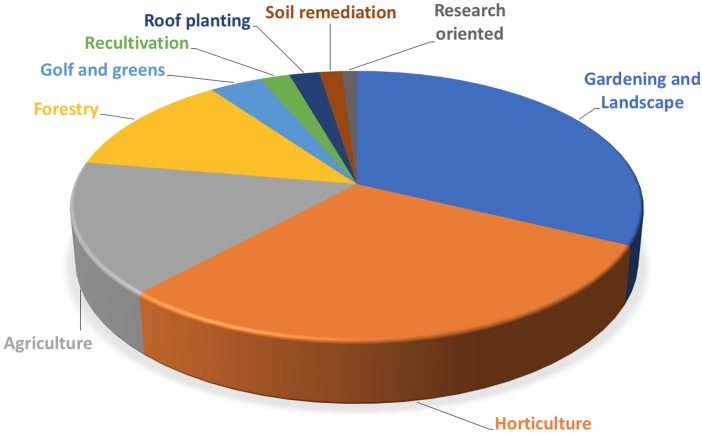
Main domains of application of AM products. The number of products of European firms was determined for each domain of application. Main fields of application are gardening and landscaping, horticulture, agriculture, and forestry.

The European market represents one of the leading markets for mycorrhizal bio-stimulants. In Europe itself, the number of firms producing and selling AMF-products has increased from less than 10 firms in the late 1990s, to more than 75 firms in 2017 (**Figure 3**). Most of the European companies are found in Germany, Italy, Spain, the United Kingdom, France, The Netherlands, Czech Republic, Austria, Belgium, Estonia, and Switzerland (**Figure 4**). The largest domains of application include gardening and landscaping, horticulture, agriculture, forestry, golf courses (in particular greens), recultivation of degraded land, roof plantings, soil remediation, and research (**Figure 5**). In terms of retail prices for hobby and semi-professional users, the average price per plant ranges between 10 and 50 cents. The cost of mycorrhizal inoculation for professional uses at an agricultural scale is considerably lower, with an estimated investment of 135 $ per hectare in the case of potato in the United States (Hijri, 2016). Apart from pure AM fungal inocula, many products include mixed fungal inocula, sometimes in combination with ectomycorrhizal fungi or with plant growth promoting rhizobacteria.

## Application of AM to Agricultural and Horticultural Crops

With the multiple benefits that AMF confer to their hosts, they hold great promise for application in crop production under various conditions. Most agricultural crops are hosts for AMF and can therefore potentially benefit from inoculation with AMF. Indeed, many studies have shown that application of commercial AMF inoculum benefits crops under agricultural conditions (Weber, 2014). Numerous studies have shown that AMF can increase plant health and yield (Mäder et al., 2000; Rouphael et al., 2015; Hijri, 2016). AMF support plant nutrition by absorbing and translocating mineral nutrients beyond the depletion zones of plant rhizosphere (see above) and induce changes in secondary metabolism leading to improved nutraceutical values. In addition, AMF interfere with the phytohormone balance of host plants, thereby influencing plant development (bioregulators) and inducing tolerance to soil and environmental stresses (bioprotector) (Rouphael et al., 2015). One important aspect of this is the promotion of root system development (Gutjahr and Paszkowski, 2013).

Since the production and application of AM fungal inoculum is relatively labor-intensive, AM application is particularly interesting for high-value crops, e.g., in horticulture, and for the adaptation of cuttings and micro-propagated plantlets in nurseries (AzconAguilar and Barea, 1997; Jeffries et al., 2003; Kleinwächter et al., 2008; Maronek et al., 2011). A large part of the horticultural plant production involves sterile micropropagation *in vitro*. A critical point of development of plantlets generated in this way is the transfer to soil (weaning) that can cause large losses (Schubert and Lubraco, 2000). Inoculation with AMF of micro-propagated fruit trees at transplant improves growth and nutrient uptake during the weaning stage, yielding plants of larger size and improved commercial characteristics (Lovato et al., 1992; Cordier et al., 1996; Schubert and Lubraco, 2000). AM fungi can accelerate this transition and improve the health of the plantlets (Vestberg et al., 2002), thereby rendering plant production more profitable. A good example for such an application are apple and peach cuttings that grow stronger with AM fungal inoculum (Schubert and Lubraco, 2000; Balla et al., 2008).

Arbuscular mycorrhiza inoculation can also be profitable in plant production at a large agricultural scale. A particularly well documented case is a large meta-analysis of potato production in 231 field trials in Europe and North America, which showed a significant increase in tuber production after inoculation with the commercial strain *R. irregularis* (DAOM 197198) (Hijri, 2016). Interestingly, in all these field trials, the farmers themselves carried out the application and evaluation under their respective conventional agricultural practice (including application of pesticides and fertilizers). This approach caused the experimental conditions to be heterogeneous, and the experimental design did not involve replicate plots or randomization. However, the large number of field sites provide robustness to the results, which were remarkably positive. Interestingly, a general beneficial effect was observed independent of location, soil type, experimentor and the details of farming practice (Hijri, 2016). The average yield increase in these 231 field trials amounted to 3.9 tons/ha, representing 9.5% of total crop yield. With an estimated threshold for profitability of 0.67 tons/ha increased yield, nearly 80% of all trials were more profitable thanks to AMF application. This impressive meta-analysis suggests that farmers of potato, and perhaps other crops, can realize significantly increased revenue thanks to AM. In addition, AMF application can allow to decrease the amount of fertilization without a decrease in yield, thereby further increasing profitability. In conclusion, such large-scale trials provide more robust results than more controlled greenhouse or small-scale trials.

Although the application of AM in horticulture and agriculture has great potential, the effectiveness and success of AMF on extended field applications depend to a large degree on external conditions that need to be taken into account. Factors such as plowing and high fertilizer application (in particular P) interfere with AMF abundance and colonization (Douds and Millner, 1999; Mäder et al., 2000; Grant et al., 2005; Hartmann et al., 2015). Other factors that affect AMF symbiosis include the use of specific biocides and cropping with non-host plants (e.g., *Brassicaceae*, *Chenopodiaceae*) (Njeru et al., 2015). In addition, for every crop, the best corresponding AM fungus should be selected (Rouphael et al., 2015), because AM fungi can provide diverse benefits (growth, stress resistance etc.), and not in each combination of plant and fungus, the trait of interest (e.g., growth) is necessarily positively influenced (Klironomos, 2003) (see above).

## Potential for the Use of AM Fungi for Renaturation, Reforestation, and Landscaping

Renaturation and afforestation are measures to stabilize degraded and eroding surfaces. In particular in arid regions, young trees are very vulnerable to abiotic stresses (drought, heat, nutrient starvation), in particular at early stages until they have established a deep root system that allows them to access ground water reserves. This critical phase can be overcome with mycorrhizal inoculation of the trees before planting. For example, the Moroccan argan tree, the fruits of which are used to prepare the precious argan oil (El Abbassi et al., 2014), are endangered in their original areas of distribution due to overuse (Lybbert et al., 2011), despite their protection as UNESCO biological reservation^2^. Argan reforestation requires that young plantlets raised in nurseries are planted out, and that they quickly adapt to the dry climate of the native range of these trees. Mycorrhizal inoculation significantly increases the growth and health of young argan trees, thereby increasing their fitness and survival after planting (Sellal et al., 2017).

A similar case is represented by the use of a mixture of indigenous AM fungi for the inoculation of young Cypress trees (*Cupressus atlantica*) (Ouahmane et al., 2007). In this study, only AM fungi isolated from the natural site of *C. atlantica* were used, thereby increasing the chances to employ fungi that are well adapted to drought and to *C. atlantica*, and avoiding to introduce new AM fungal species with unpredictable effects on the local environment. AM inoculation not only increased plant growth, but also increased survival of the trees in the dry native conditions. This latter point is perhaps even more important than the growth promotion, because it renders reforestation efforts more sustainable.

Another interesting example is stabilizing sand dunes by planting of the drought-tolerant mesquite tree (*Prosopis juliflora*), which increases mycorrhizal communities in sand dunes (Moradi et al., 2017). On the other hand, the mesquite trees profit from AM colonization (Solis-Dominguez et al., 2011). Hence, AM symbiosis can be a critical component in strategies to protect vulnerable sandy soils against erosion, and to improve their fertility.

## Can AM Fungi Promote Bioremediation of Contaminated Soils?

During the last decades, the potential of plants has been explored to reduce the contamination of soils polluted by organic compounds or heavy metals, and AM fungi could potentially play a central role in such strategies (Leyval et al., 2002; Turnau et al., 2006; Khade and Adholeya, 2007; Sheoran et al., 2010). Thanks to their mineral-scavenging capacities, and with their protective role against abiotic stress, AM fungi can potentially promote plant growth in contaminated soils, a capacity commonly referred to as bioremediation (Leyval et al., 2002; Göhre and Paszkowski, 2006). They can do so in two ways: they can either accumulate and sequester toxic metal ions, thereby protecting their host from the pollutant (Weissenhorn et al., 1995; Diaz et al., 1996; Gonzalez-Chavez et al., 2004), or they can deliver them to the host just like essential mineral nutrients such as Cu and Zn, resulting in heavy metal accumulation in the host. In the first case, plant production can be enabled in polluted substrate, with minimal contamination of the crop. In the second case, the plants can be harvested and destroyed to reduce the heavy metal load of the site (phytoextraction) (Burns et al., 1996; Khan et al., 2000). Of course, both approaches require heavy-metal-tolerant AM fungi, and phytoextraction in addition requires highly tolerant host plants that can cope with toxic heavy metals, and at the same time yield large shoot biomass in order to accumulate significant amounts of heavy metals.

To date numerous laboratory studies have been carried out to explore the potential of AM in bioremediation of the soil, however, only few field studies have addressed the applicability of this approach to large scale conditions (Burns et al., 1996; Adriano et al., 2004; Chibuike, 2013). Worldwide, there are only few companies offering AM fungal products for bioremediation. Some of the obstacles include the fact that most heavy metal-accumulating plants are rather small, and some are not host plants for AM fungi (e.g., the crucifer *Thlaspi*). In addition, AM colonization is often reduced by high pollution.

## Can Am Fungi Be Bred for Improved Symbiotic Traits?

Given the promising features, but also the limitations of AM fungi for application in plant production, it would be a great asset if they could be bred for higher efficiency in the respective context of interest. Classical breeding (by crossing and selection in the progeny of variants with new desired features) is currently impossible with AM fungi owing to their particular genetic constitution (Sanders and Croll, 2010). Their syncytial nature and their purely clonal propagation, as well as the absence of recognizable sexual stages prevents forward and reverse genetic approaches such as mutant screening, transformation, crossing, genetic mapping etc.. However, the mycelia of AM fungi can fuse by a process known as anastomosis, which allows for the exchange of genetic material (incl. nuclei) between the two syncytia (Giovannetti et al., 1999).

“Crossing” of AM fungi by anastomosis, and subsequent culturing of AM fungal progeny can generate new genotypes with new symbiotic features (Angelard et al., 2010; Angelard and Sanders, 2011). In particular, new beneficial mycorrhizal traits can result from this kind of breeding scheme (Angelard et al., 2010). However, anastomosis is only possible between compatible AM fungal isolates of the same, or perhaps closely related species (de Novais et al., 2017), indicating that it requires genetic compatibility factors. Based on these findings, AM fungi could potentially be bred for improved symbiotic traits by systematic genetic reshuffling between divergent (but compatible) AM fungal isolates, followed by screening for the most beneficial new strains among AM fungal segregants. This screening should be performed with each host plant of interest, thus allowing to identify the best-suited AM fungal segregant for each target host species. Such combinations could subsequently further evolve by continued selection for improved AM fungal descendants with more beneficial effects on the host plant. Such changes can emerge surprisingly quickly, possibly driven by genetic drift among the heterogeneous nucleotypes of the expanding syncytial AM fungal mycelium (Angelard et al., 2014).

## Future Opportunities and Challenges for AMF

Inspite of its growing trend, the current market for mycorrhizal products remains far from its full potential. Apart from technical issues, challenges for AM fungal products in the coming years include (i) political and regulatory constraints; (ii) quality assurance and product efficacy; and (iii) customer awareness and acceptance.

In terms of regulations and policies, the current market for mycorrhizal products has, to date, remained relatively unrestricted by political forces. In Europe, there is no unifying regulation covering (and controlling) the manufacture, use or movement of mycorrhizal fungal products (Vosatka et al., 2008). Depending on their intended use, AM fungal products could be registered in the market in three different categories: as bioprotectants, as biofertilizers, or as biostimulants. The registration has to be performed according to the national regulations of each EU state member. In some cases (e.g., France and Belgium), the regulatory process is quite complex and expensive. Such regulations result in limitations and market entry barriers for AM fungal products (Vosatka et al., 2008). In this regard, the European Parliament is currently evaluating the establishment of an equitable EU market for biostimulants. The key elements considered by the EU to establish a single-market include: defining biostimulants and defining the boundary with plant protection products; the requirement to develop safety criteria and harmonized standards, in particular for microorganisms, and promoting a circular economy with the efficient use of plants and plant extracts. A single harmonized market for biostimulants will support EU farmers to become more competitive and participate in developing sustainable agriculture with a reduced impact on the environment.

In relation to product quality, given the lack of regulatory bodies to set the quality parameters, AMF producers rely on self-imposed quality standards to ensure best practice in production (Vosatka et al., 2008). In Europe, for instance, the main AMF producers have agreed on the use of a protocol proposed by Gianinazzi-Pearson et al. (1985) to define quality of AMF products. This protocol is known as the “most probable number” (MPN) and serves to determine the presence or absence of AMF in a dilution series, with the results interpreted as a probability estimate of propagule number from a statistical table. Even though the assay is indirect (absolute numbers of propagules are not measured), it has the advantage of providing a single number that can be compared directly with other tests in the same assay. However, other qualitative parameters should also be taken into account, in particular richness of inoculum (number of spores or propagules/ml) and infectivity, i.e., the capacity of the inoculum to establish mycorrhizal symbiosis. Finally, not every combination of a plant and an AM fungus is beneficial (see above), hence, it is advisable to test different AM inocula for each crop of interest to identify optimal combinations of plant and AMF.

In general, product quality and efficiency are still areas that require further attention. The appropriate dosage, or propagule density, for a given market sector is not yet formalized and it leaves scope for the marketers to set these values. The aforementioned constraints open a window of opportunity for the research community in order to assist producers and the market in defining what should be the minimum treatment standard (Vosatka et al., 2008). Other critical challenges for the AM market are customer awareness and acceptance. Although the use of biostimulants and biofertilizers is growing in popularity, the use of traditional chemical fertilizer products remains as the most common practice among farmers. In this respect, AMF producers are focusing their efforts to establish relevant case studies and field trials to demonstrate and prove the benefits of AMF in agriculture and horticulture. Larger organisms contributing to the promotion of biostimulants include the The European Biostimulant Industry Council (EBIC^3^), and the International Mycorrhiza Society (IMS^4^).

## Outlook

Arbuscular mycorrhizal fungi promote many aspects of plant life, in particular improved nutrition, better growth, stress tolerance, and disease resistance. In addition, the hyphal networks of AM fungi improve soil characters such as soil particle aggregation thereby improving the resistance of soil toward erosion by wind and water. Finally, AM fungi decrease nutrient leaching from the soil, thereby contributing to the retention of nutrients in the soil, and decreasing the risks of contamination of ground water. These multiple benefits of AM fungi translate into significant ecological services in natural contexts. The promises for agriculture have been clearly documented for certain crops, in particular potato, however, many applications have still to be developed, which requires significant investment in research and development of AM fungal inocula suited for additional crops.

## Author Contributions

MC, MA, LB, and EN contributed significant parts of the text. DR was the main coordinator and wrote a large part of the manuscript.

## Conflict of Interest Statement

The authors declare that the research was conducted in the absence of any commercial or financial relationships that could be construed as a potential conflict of interest.

## Footnotes

1 http://www.teriin.org

2 http://www.unesco.org

3 http://www.biostimulants.eu

4 http://mycorrhizas.org
